# The Development of Military Orthopedie Surgery under Colonel Sir Robert Jones

**Published:** 1918

**Authors:** 


**The Development of Military Orthopedic Surgery under Colonel Sir Robert Jones.** Major Robert B. Osgood, M. R. C., Consultant in Orthopedic Surgery, A. E. F., presented a paper in which he expressed regret that Colonel Sir Robert Jones could not be present to tell the " almost romantic " story of British military orthopedic surgery as it had developed under his leadership. At the beginning of the war the need for orthopedic centers was only vaguely conceived, even by Sir Robert himself. He was placed in charge of a military hospital for bone and joint cases at Alder Hey near Liverpool, but soon after was given the title of Inspector of Military Orthopedics and took the first steps towards the creation of a bone and joint centre at Shepherd's Bush (London), to be developed as the need for special care and treatment in these cases and their increasing number and importance became manifest. The Liverpool and London centers are to-day the most influential and among the largest of the orthopedic hospitals.

The necessity for providing a large number of beds in Great Britain for soldiers more or less crippled arose from three main causes : —

1st. The inevitable maiming which gun-shot wounds themselves caused as a result of serious damage to joint structures, loss of substance of bone and muscle tissue, and complete severance or loss of conductivity of the peripheral nerves.

2nd. The secondary changes in bone, muscle, and nerve tissue, which resulted from the universal infections occurring in these wounds in the early part of the war.

3rd. The lack of preventive treatment accorded these cases before and after they reached the General Home Hospital.

The definition of what constituted an orthopedic case was made very broad and inclusive. It covered mal-united and ununited fractures, all femoral fractures, old injuries to nerves, cases needing muscle transplantation, those requiring special surgical appliances (including artificial limbs), and, in general, all deformities of bones and joints.

To deal with these conditions many new centres were established, while the old ones increased their number of beds or took over whole hospital plants. Necessary operations represented only a part of the activity of these centres. Departments of massage, electrical and hydrotherapeutic treatment were organized and equipped with trained lay and medical personnel and with complicated and expensive apparatus.

It soon became evident that curative occupation was almost an essential for the speedy recovery of these groups of discouraged men; it also aided in maintaining discipline, and men who had constantly evaded rules began to obey them, those who had overstayed their leave and returned drunk came back on time and sober, while those who had refused operations, calculated to give them more function, sought them. Workshops became a helpful part of every center.

*Present Organization.* The commanding officer of the hospitals in which these centers are situated is in direct charge of their administration, passing up through the A. D. M. S. and the D. D. M. S. to the Director General of Medical Services. The professional work is in charge of an orthopedic surgeon or a general surgeon especially interested in bone and joint work. In direct connection with each hospital is a consulting surgeon, to whom the staff have direct approach, and who may take matters up with the Inspector of Orthopedic Surgery, Sir Robert Jones, or other officers. Reports show that from 75 to 85 per cent. of the men are returned to the army in one category or another — an unexpectedly high percentage in view of the type of cases.

*Further development.* The necessity of standardized splinting at the earliest possible moment has become evident. The front line splint drills have been adopted in most of the British armies; they are recognized as pain saving, shock preventing, and even life saving measures. Both the French and British realize that the establishment of special centers for the treatment of fractures is essential, and the Americans must do the same. In amputated cases, weight bearing must begin at the earliest possible moment that the condition of the stump will allow. It is better for the physical condition of the stump and the mental condition of the soldier, and prevents the muscular contraction which makes later fitting of apparatus impossible until it is overcome. Experience has shown that from one-third to one-half of the ordinary deformities resulting from gun-shot wounds are preventable. Though the ability to exercise preventive measures is never a question of minutes it is often a question of a few days. There is a psychological moment in convalescence when the soft callus of a bowing bone is mouldable to proper alignment, when a contracting joint may be gradually and safely straightened without suffering or operation. The demand for speedy evacuation from the temporary hospitals where the greater part of the surgery is done focuses attention on the immediate results. The surgeons upon whom, in the various later stages of convalescence, the care of the case devolves, feel a certain lack of responsibility for the functional result of the first operation, and devote their chief attention to the general condition of the patient and the healing of his wound, since the pressure for beds in these general hospitals is only slightly less than in the front lines.

Surgeons must free themselves from their tendency to treat the *wounds* and forget the *function*; to make a *well* man but not a *working* one; to take the anatomical rather than the physiological point of view.

